# Aflatoxin levels in maize and peanut and blood in women and children: The case of Timor-Leste

**DOI:** 10.1038/s41598-019-49584-1

**Published:** 2019-09-11

**Authors:** Luis de Almeida, Robert Williams, Dirce M. Soares, Harry Nesbitt, Graeme Wright, William Erskine

**Affiliations:** 1 AI-Com, Ministry of Agriculture and Fisheries, PO Box 221, Comoro-Dili, Timor-Leste; 2 Centre for Plant Genetics and Breeding, UWA School of Agriculture and Environment & Institute of Agriculture, University of Western Australia, 35 Stirling Highway, Crawley, WA 6009 Australia; 3 Ministry of Health, PO Box 374, Dili, Timor-Leste; 4 Peanut Company of Australia (PCA), 133 Haly Street, Kingaroy, Qld 4610 Australia

**Keywords:** Plant ecology, Secondary metabolism

## Abstract

Aflatoxins are toxic fungal metabolites produced by *Aspergillus* sp. with carcinogenic properties that are a common food contaminant of many crops including maize and peanuts. In Timor-Leste malnutrition and children’s stunting are frequent and maize and peanuts are staple foods. This study aimed to provide information on aflatoxin exposure nationally. The study measured levels of aflatoxin in locally-produced maize and peanuts (296 samples) and of aflatoxin-albumin conjugate in blood samples of women and young children (514 and 620 respectively) across all municipalities. The average concentration of aflatoxin in the grain samples was low with most maize (88%) and peanut (92%) samples - lower than European Commission tolerated aflatoxin level. Although aflatoxin–albumin conjugate was detected in more than 80% of blood samples, the average concentration in children and adults of 0.64 and 0.98 pg mg^−1^ alb, respectively, is much lower than in other similar rural-based countries. Although low in concentration, blood aflatoxin levels and aflatoxin contamination levels in maize across municipalities were correlated significantly for mothers (R^2^ = 37%, n = 495) but not for children (R^2^ = 10%). It is unlikely that the consumption of aflatoxin contaminated grain is a causative factor in the current level of malnutrition and stunting affecting Timor-Leste children.

## Introduction

Aflatoxin-affected grain presents a major global health issue to both commercial and subsistence farming. Estimates from the Food and Agriculture Organization of the United Nations (FAO) are that ~25% of cereals produced in the world are contaminated by mycotoxins^1^. In developed economies contaminated food products have resulted in great economic losses worldwide^2,3^. This is often a serious constraint for grain producing countries where the contamination of raw products such as maize, peanut and pistachio kernels may lead to consignment rejection^4^.

Specific regulations related to aflatoxin contamination have been implemented by several countries to minimize the risk in food products – particularly maize and peanuts^2,5–7^. For aflatoxin the guidelines to regulate the levels of its exposure relate to human food and animal feed^8^.

Most developing countries, where subsistence farming predominates, do not or cannot regulate the quality of grain consumed by the population. Subsistence farmers, who both produce and consume their own produce, are especially exposed to the negative impacts of aflatoxin affected grain, as it is not subject to quality testing.

Aflatoxins have been widely recognized as a group of chemically similar, toxic fungal metabolites produced by *Aspergillus* sp.^3,5,9^. Although 18 types of aflatoxin have been found, only four (aflatoxins - B1, B2, G1 and G2) are recognised as common contaminants of food products^10,11^. Aflatoxins affect the health of both humans and animals when ingested at a high concentration and they are classified as strong carcinogens (Group 1) by the International Agency for Research on Cancer^12–15^.

The filamentous fungus *Aspergillus* can grow on grains before harvest and in storage to contaminate a wide range of food crops including maize, peanuts, nuts, spices, fruits and their products^4,6,16^. Among such hosts of the fungus, maize is the most important food staple in Timor-Leste and peanuts provide a major source of protein to the population’s diet^17–19^. Both crops are cultivated in a tropical environment conducive to aflatoxin contamination with temperatures ranging from 25–35 °C. The rural population of Timor-Leste predominately comprises subsistence farmers, who generally eat what they produce, so contamination of maize and peanuts with aflatoxin will rapidly enter people’s diets. Many of the population are malnourished and consequently the 2015 Global Hunger Index ranked the country as the fourth lowest globally, and as one of only three countries where more than 50% of children under age five suffer from stunting^20^. One factor that could potentially contribute to growth retardation in children is exposure to aflatoxin^21^.

The only prior information on aflatoxin incidence in Timor-Leste comes from a small survey to validate the Aflatoxin QuickTest^TM^ ^15^. The study described here examined aflatoxin presence in maize and peanuts grown in Timor-Leste. As measuring aflatoxin-albumin levels from blood samples is an appropriate method to measure exposure to aflatoxins over the past weeks/months, aflatoxin exposure was assessed through analysis of blood samples from children and their mothers^22^. The data obtained permitted an overview of the aflatoxin issue in Timor-Leste.

## Results

Aflatoxin in grain samples. Aflatoxin was detected in 36% of the maize samples across the three collection years (Table 1) with an average contamination of 11.4 ng mL^−1^ (SD = 50.5). Of the grain with detectable levels, most were low (24.5%) and below the EC permissible maximum level of 15 ng mL^−1^ ^23^. Across the three years only 11.6% of maize samples had aflatoxin levels above 15 ng mL^−1^. There was no difference in the level of contamination between the years (P = 0.116), so further analysis was done with data of all three years combined. The highest level of aflatoxin detected was 573 ng mL^−1^. Maize sampled from food storage in farmers’ houses had lower levels of aflatoxin (P = 0.028) than maize samples sourced from markets and from seed producers (Table 2).

**Table 1:** Number of grain samples collected per year of maize and peanut from different sources (seed producers, markets and households), mean level of aflatoxin contamination (log (1+ aflatoxin level (ng mL^−1^))) and the percent of maize and peanut samples with non-detectable, low, high and very high aflatoxin levels across the years.

Species	Year	Seed producer	Market	House-hold	Total	Mean level (log (ng mL^−1^ + 1))	% Non-detectable (<2 ng mL^−1^)	% Low (2–15.0 ng mL^−1^)	% High (15.1–100 ng mL^−1^)	% Very high (>100 ng mL^−1^)
Maize	2013	27	53	0	80	0.35	63.8	28.8	3.8	3.8
	2014	15	38	27	80	0.31	75.0	11.3	11.3	2.5
	2015	4	25	1	30	0.33	53.3	33.3	10.0	3.3
Mean over years						0.33	64.0	24.5	8.4	3.2
Peanut	2014	16	65	0	81	0.31	84	11	2.5	2.5
	2015	3	22	0	25	0.32	64	16	12	8
Mean over years						0.31	74	13.5	7.2	5.3
Total		65	203	28	296

**Table 2:** The mean level of aflatoxin contamination (log (1+ aflatoxin level (ng mL^−1^))) and the percentage of maize and peanut samples with non-detectable, low, high and very high recorded for different sources (houses, markets and seed producers) across the years.

Source	Mean level (log 1 + ng mL^−1^)	% Non-detectable (<2.0 ng mL^−1^)	% Low (2–15.0 ng mL^−1^)	% High (15.1–100 ng mL^−1^)	% Very high (>100 ng mL^−1^)
**Maize**					
Household storage	0.08	96	0	0	4
Markets	0.43	61	27	8	4
Seed producers	0.39	66	26	4	4
L.S.D. (P < 0.05)	0.13				
p value	0.028				
**Peanut**					
Market	1.4	75	17.8	3.6	3.6
Seed producers	1.7	90	0	5	5
p value	0.65				

Aflatoxin B1 was the most commonly detected aflatoxin found in 32.1% of samples and had the highest concentration (Table 3) with a geometric mean level of 9.5 ng mL^−1^. Whereas, B2 was found only in samples contaminated with B1 at approximately half the frequency. Aflatoxins G1 and G2 were present at still lower incidence. The levels of G1 and G2 were not correlated with the levels of B1 and B2 toxins. G1 and G2 were sometimes present at levels similar to B1 and B2, but were absent in many samples, including those samples with a high level of B1 and B2.

**Table 3:** Frequency detection of aflatoxins B1, B2, G1 and G2 and geometric mean level of aflatoxin in contaminated maize and peanut samples.

Type of Aflatoxin	Maize		Peanut	
	Incidence of infected samples (%)	Geometric mean of aflatoxin of infected samples (ng mL^−1^)	Incidence of infected samples (%)	Geometric mean of aflatoxin of infected samples (ng mL^−1^)
B1	98	9.5	90	8.2
B2	34	3.6	45	3.2
G1	10	1.7	69	2.9
G2	2	1.0	45	0.5

Regarding peanuts, aflatoxin was detected in 26% of the samples collected over the two years (Table 1) with an average contamination of 12.8 ng mL^−1^ (SD = 52.0). With a contamination level below 15 ng mL^−1^ considered acceptable for human consumption, 87.5% of the samples tested were safe as human food and the only 12.5% of samples registered levels above 15 ng mL^−1^. With no difference in mean contamination levels between the two years (P = 0.432), the data were combined for further analysis. There was no difference (P = 0.65) in the level of contamination between peanuts sold for consumption and peanuts produced for seed (Table 2). The average contamination level for all samples tested was 12.8 ng mL^−1^.

As with maize, aflatoxin B1 was the most common aflatoxin detected in peanut (25% of samples) and had the highest concentration with a geometric mean level of 8.2 ng mL^−1^ (Table 3). B2 type was found only in samples contaminated with B1 at about half the frequency of its concentration. G1 was present in 18% of the samples, with a geometric mean of 2.9 ng mL^−1^. Whereas, G2 was only present with samples contaminated with G1 and was the aflatoxin with the lowest concentration among the four types assessed. The level of G1 and G2 was not correlated with the levels of B1 and B2. G1 and G2 were sometimes present at levels similar to B1 and B2, but were absent in many, including samples that have a high level of B1 and B2 (Supplementary Table S1).

### Aflatoxin in blood samples

**Figure 1:**
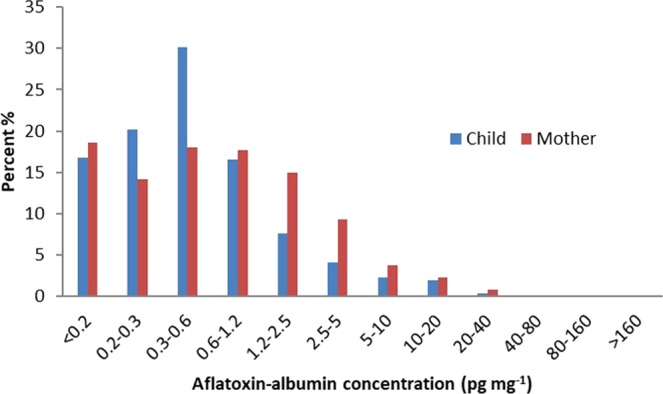
Distribution (% of samples) of Afla-alb concentrations (pg mg^−1^ alb.) of children and their non-pregnant mothers in Timor-Leste.

Using the data set available from blood serum collection to detect aflatoxin concentration, a total of 514 children and 620 mothers were analysed for both children aged between 6 to 59 months and their non-pregnant mothers (age range from 14 to 60). The distribution of Afla-alb concentrations of children and non-pregnant mothers is shown in Fig. 1. For children 86 samples (17%) were detected with a concentration of <0.20 pg mg^−1^ aflatoxin albumin adducts, 428 samples (83%) were > = 0.20 pg mg^−1^ alb. with a range from 0.20 to 37.0 pg mg^−1^ alb. For children the geometric mean value of B1-lysine aflatoxin albumin adduct was 0.64 pg mg^−1^ alb.

For non-pregnant mothers, 115 samples (19%) were identified with a concentration level of <0.20 pg mg^−1^ blood albumin, and 505 samples (81%) had a concentration level of > = 0.20 pg mg^−1^ blood alb. (range 0.20 to 179.3 pg mg^−1^ alb.). In non-pregnant mothers, aflatoxin albumin adducts showed a geometric mean value of 0.98 pg mg^−1^ alb.

Regarding correlations, the blood aflatoxin albumin concentration in the mother was weakly correlated with her child’s aflatoxin albumin levels (R^2^ = 17.4%). Correlations between child aflatoxin albumin level and indicators of mal-nutrition (blood haemoglobin level, z score for height for age, weight for age and weight for height) were all non-significant (P < 0.05) (Supplementary Table S2). Correlations between mother blood aflatoxin albumin level and indicators of mal-nutrition were also non-significant (P < 0.05). Male and female children had the same level of aflatoxin albumin level, and similarly there was no difference in blood levels between children in rural and urban areas.

### Spatial analysis

**Figure 2:**
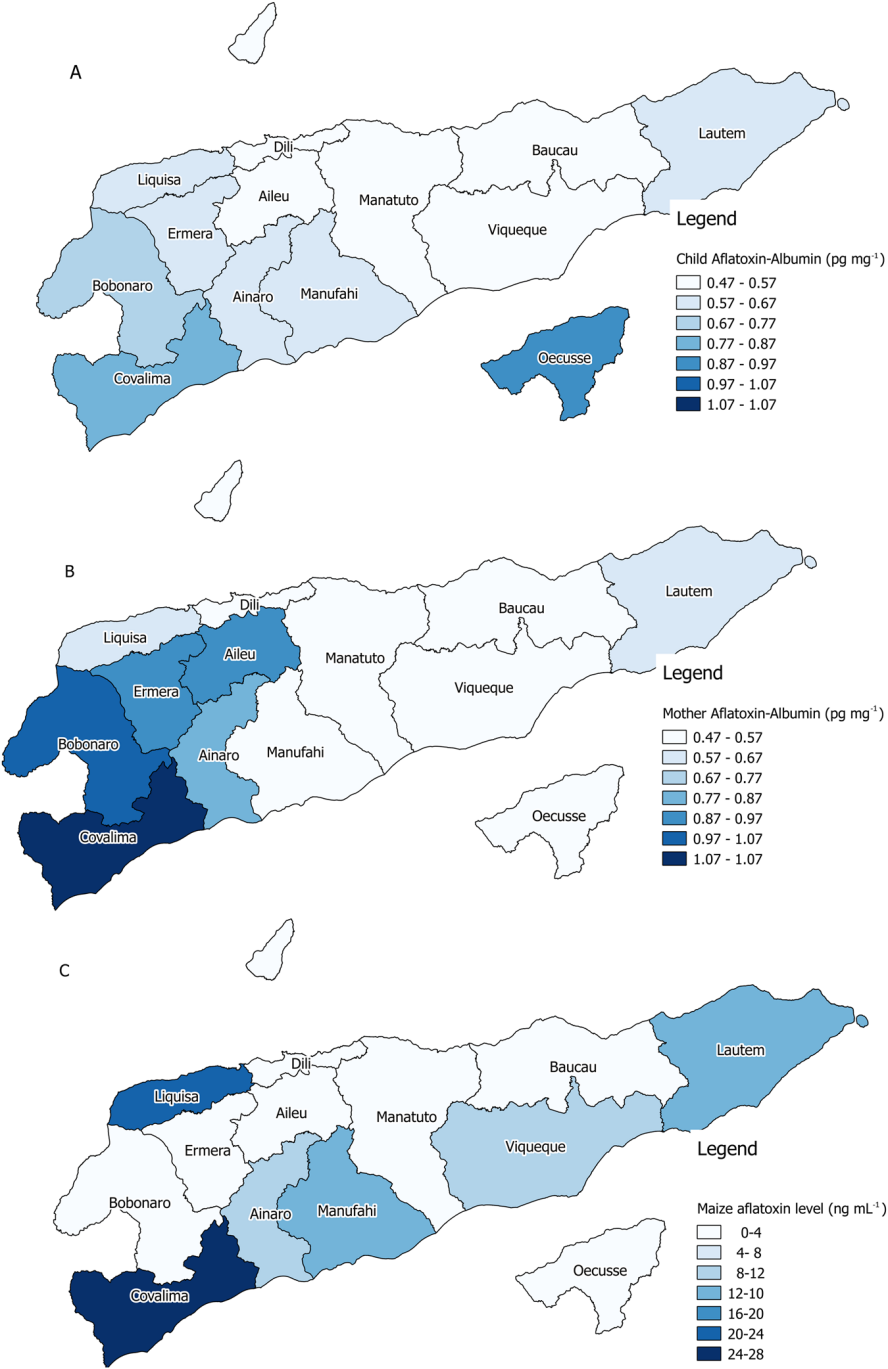
Aflatoxin level by municipality for (**A**). Child blood, (**B**). Mother blood and (**C**). Maize grain. The exclave of Oe-cusse is excluded due to insufficient number of samples collected.

Aflatoxin contamination of maize samples was generally worse in the south coast, especially in western municipalities and the eastern municipality of Los Palos (Fig. 2). For peanuts differences between municipalities in aflatoxin contamination were non-significant (P < 0.05). Blood aflatoxin levels in mother and children were highest in the mainland western municipalities and in the far eastern municipality of Los Palos (Fig. 2). Lower blood aflatoxin levels were recorded in the central districts of the country. Municipality mean blood aflatoxin levels and aflatoxin contamination levels in maize were correlated significantly (P < 0.05) for mothers (R^2^ = 37%) but not for children (R^2^ = 10%, n = 10).

## Discussion

Aflatoxin contamination of food products is a global health concern. Maize and peanuts are common hosts of mycotoxin producing *Aspergillus* sp. and in Timor-Leste both crops are food staples with maize the most important crop nationally^21^. Consequently the first national assessment of aflatoxin levels in these grains is important.

Of the three sources for the maize sample (households, markets and seed producers), samples purchased directly from households were less contaminated than samples sourced from local markets and seed producers. We anticipated the contrary i.e. that samples collected from commercial seed producers would have a lower contamination rate, as the commercial seed farmers are paid a premium for drying grain quickly and storing the dry grain appropriately. Both measures would assist in reducing aflatoxin contamination post-harvest. But found them to be more contaminated than those samples sourced directly from households. This reflects that the household food supply is carefully selected to become food for the family. As maize cobs are threshed or brought into the storage areas, they are checked for freedom from moulds and other deformations. It appears that this selection of clean cobs for food produces a food supply rarely contaminated with aflatoxins. In contrast, commercial seed is produced on a larger scale, and may not be selected as rigorously for freedom of visible ear rots and disease. Maize grain sold in markets is mostly produced and transported from other locations, therefore contamination can easily occur during transportation. Moreover, the markets in Timor-Leste are mostly open air with a low standard of storage facilities. Mutegi *et al*.^24^ stated that moving products from one location to the other and in combination with inappropriate storage facilities in Western Kenya is known to favour aflatoxin contamination. Clearly seed producers and traders need training to increase their awareness of aflatoxin contamination and management.

For comparison with these results from Timor-Leste, a number of countries particularly those with similar studies on maize grain were selected (Table 4). In terms of the rank for the percentage of aflatoxin contamination, China^25^, Serbia^26^, Kenya^27^ and Brazil^28^ ranked higher than Timor-Leste (Table 4). Considering the maximum levels of aflatoxin detected, the highest level was found in Eastern/Central Kenya^25^ (46,400 ng mL^−1^) followed by in China-Guangxi^23^ (2,496 ng mL^−1^), in contrast in Timor-Leste we found the highest detectable level of aflatoxin was 573 ng mL^−1^. The intermediate level in Timor-Leste is probably due to good drying environments soon after harvest of maize and peanuts, that allow for rapid drying of the grain, limiting the time for fungal growth and possible contamination. The intermediate levels of aflatoxin contamination measured in Timor-Leste also reflects the subsistence level of food production in the country. Subsistence farming is where the food grown is mostly eaten by that farming household and is rarely sold. As there is no centralised collection and processing, it is not possible to sort and segregate contaminated grains and pulses. Developed countries, such as USA and Canada, have regular screening and separation of contaminated grain, resulting in very low levels of aflatoxin. It appears therefore that the level of aflatoxin contamination in Timor-Leste is similar to that of other subsistence farming nations. In short, in the first national study of aflatoxin in maize grain, the levels in Timor-Leste could be classed as intermediate compared to other maize growing countries. Notwithstanding this aflatoxin still represents a risk in the production and consumption of maize that requires management locally.

**Table 4:** Incidence of aflatoxin occurrence in maize grain and peanut kernels of this study and selected countries.

Country	Maize			Peanut		
	Positive samples (%)	Contamination range (ng mL^−1^)	Mean/median contamination rate (ng mL^−1^)	Positive samples (%)	Contamination range (ng mL^−1^)	Mean/median contamination rate (ng mL^−1^)
Brazil^28,31^	42	0.05–8.3	n/a	51	43–1099	n/a
China^25,34^	85	9–2,496	n/a	25	0.1–703	2.1
Egypt^33,59^	33	n/a	9.8	82	n/a	n/a
Kenya: E. & Central^27^	55	1–46,400	20.5			
Kenya: Western^24^	49	n/a	2.3			
Malaysia^30^				78	0–103	11.2
Pakistan: Punjab^3^	35	12.1	n/a			
Serbia^26^	68.5	1.01–86.1	36.3			
Taiwan^60^				7.8	n/a	14.9
Tanzania^5,61^	18	0.01–158	24			
Timor-Leste	36	2.1–573	11.4	26	2.1–365	12.8
Turkey^62^	n/a	0.01–32.3	10.9			
Zambia^63,64^	21.4	0.7–108.4	n/a	55.4	0.01–48.7	0.4
Zimbabwe^32^				17	6.6–622.1	n/a

The level of aflatoxin contamination in maize along the south coast (Fig. 2) is associated with the bimodal rainfall pattern in this part of the country, where two maize crops a year can be produced^29^. Maize grown in the rainfall peak of December-April is often drying when the next rains start in May. Poor drying conditions for the main crop may lead to higher contamination rates along the south coast and the eastern municipality of Los Palos, which also has a bimodal rainfall pattern.

In peanuts, aflatoxin was detected in 26.0% of all peanut kernels sampled. This comprised half (13.5%) of the detectable samples with levels below the tolerable level of 15 ng mL^−1^ (below the legislative limit established by EC), and the remaining half - 12.5% of all samples - with levels above 15 ng mL^−1^. Clearly, aflatoxin-infected kernels represent a risk to peanut production and consumption in Timor-Leste, where the majority of farmers produce food crops for their own families. Awareness needs to be increased of the aflatoxin risk in peanuts and of techniques to reduce contamination. This study was on peanut kernels (raw product only). Peanuts are used as a base for several other food products. Research is required on the occurrence and levels of aflatoxin in peanut products in the country, and how to avoid contamination at harvest and/or during post-harvest processing.

For an international comparison, seven countries with similar studies for the assessment of peanut aflatoxin contamination were selected (Table 4). The percentage of samples with positive aflatoxin detected showed that four countries - Egypt, Malaysia, Zimbabwe and Brazil - were higher than Timor-Leste^30–33^. The maximum contamination level for peanut obtained in Timor-Leste was 365 ng mL^−1^ which indicates that the risk of peanut aflatoxin was considerably below the maximum levels recorded in Brazil (1099 ng mL^−1^), China (703 ng mL^−1^) and Zimbabwe (622 ng mL^−1^)^31,32,34^. This study as the first national assessment on peanut aflatoxin in Timor-Leste will guide decisions on food safety and regulation.

Among the four types of aflatoxin assessed (B1, B2 G1 and G2), B1 aflatoxin was the most commonly detected in both maize and peanuts in Timor-Leste. This confirms the findings of a number of authors - as reviewed in Enyisi *et al*.^35^ – that the B1 aflatoxin is the most common type elsewhere. The B1 type has been reported as high risk for a number of health problems and economic losses due to market rejection.

The low level of aflatoxin contamination in maize and peanuts could be due to two factors, one is the low level of field contamination, and secondly fungal-affected maize cobs and peanut kernels are generally sorted out by farmers and family members. Farmers should be encouraged to (1) dry their corn and peanuts as quickly as possible, (2) continue to select clean cobs and pods to be used as food and (3) store produce in dry places.

However, with the development of food based industries in Timor-Leste there will be a need to detect the approximately ten percent of samples that have levels of aflatoxin above the EC food standard. A new aflatoxin Quicktest developed by Sánchez-Bayo *et al*.^15^ has recently been implemented in Timor-Leste, allowing food processors to confidently purchase maize and peanuts that are free of aflatoxin. The QuickTest survey used maize and peanut samples from one year to calibrate an ELISA-based Quicktest for maize and peanuts.

This study importantly covered aflatoxin levels in Timor-Leste for both grain and blood samples of children and mothers/adults. AF-Albumin levels in blood samples have been demonstrated as a promising approach to detect exposure to aflatoxin^36^. The method has been used in a wide range of countries as a measure of exposure (Table 5). Assuming the half-life of aflatoxin-lysine is 20 days, the blood test gives a measure of exposure for the past weeks/months. The other approach of urine testing for metabolites of aflatoxin has the limitation that it is likely to reflect exposure only for the last 24–48 hours. Considering the children’s data for aflatoxin B1-lysine, the geometric mean values for the level of the adduct (0.64 pg mg^−1^ alb.) from this study were much lower compared to those results from other selected developing countries such as Benin and Togo with a geometric mean value of 32.8 pg mg^−1^ alb.^37^, Tanzania 12.9 pg mg^−1^ alb.^38^, Gambia 10.1 pg mg^−1^ alb.^39^ and in Guinea 9.9 pg mg^−1^ alb.^40^ (Table 5).

**Table 5:** Incidence of aflatoxin-albumin (pg mg^−1^ alb.) in children and adults/mothers blood samples in this study and selected countries.

Country	Child/Adult	Range of age	Positive samples (%)	Geo. mean Afla-alb level
Benin & Togo^37^	Children	9 to 60 mth	99	32.8
Brazil^45^	n/a	n/a	62	10.7
Canada^48^	n/a	n/a	(nd) 0	(nd) 0
Egypt^45^	Adult (male/female	36 to 74 yrs	67	5
France & Poland^45^	n/a	n/a	0/74 (0)	0
Ghana^44^	Adult/mother	14 to 48 yrs	40	5
Guinea^40^	Children	2 to 5 yrs	96	9.9
Kenya^43^	Pregnant/with child >24 mth	n/a	n/a	7.5
Malaysia^46^	Adult male/female	18 to 85 yrs	97	4.5
Tanzania^38^	Children	12 to 22 mth	84	12.9
The Gambia^3,39,41^	Adult (male/female) Rural	<15 to >35 yrs	100	61.8
	Mother	n/a	100	40.4
	Adult (male/female) Peri urban	<15 to >35 yrs	98.8	39.1
	Children	1.4 yrs	11	10.1
Timor-Leste	Adult (non-pregnant)	14 to 60 yrs	81	0.98
	Children	6 to 59 mth	83	0.64
Turkey^47^	Adult (male/female)	17 to 64	86	1.1
USA^48^	n/a	n/a	1.2	0.3

n/a, non-available; nd, non-detectable.

Aflatoxin albumin adducts in mothers/adults measured in this study also revealed that the level found in Timor-Leste had a geometric mean of 0.98 pg mg^−1^ alb., which is considerably lower than levels reported from Gambia with a geometric mean value of 40.4 pg mg^−1^ alb.^39,41^ with the geometric mean values of 61.8 pg mg^−1^ alb. and 39.1 for both rural and peri-urban samples, respectively. Most of the other selected developing countries such as Brazil^42^, Kenya^43^, Ghana^44^, Egypt^45^, Malaysia^46^ and Turkey^47^ had lower geometric means than in Gambia; but such means were all greater than the means for both children and mothers/adults from Timor-Leste. Among the selected countries Timor-Leste only shows a geometric mean slightly greater than those of developed countries - USA (0 pg mg^−1^) and Canada (non-detectable)^48^; and France and Poland (0 pg mg^−1^)^45^.

Among municipalities, mother blood aflatoxin level in the central western municipalities of Aileu, Emera and Maliana were much higher than expected from the maize contamination level. This is unsurprising as maize produced in the south west municipality of Suai is often traded and sold in these three districts. It is likely that contaminated maize in Suai is being sold and consumed in the neighbouring inland districts of Aileu, Maliana and Emera, which do not have a second maize crop.

The incidence of aflatoxin albumin measured in blood samples of children and adults/mothers revealed that ˃80% were positive in Timor-Leste. Considering adults, blood aflatoxin albumin levels detected in 81% of the samples were very similar to other subsistence farming countries, and much lower than developed countries. However the mean value of level detected was much lower than those reported in other developing countries (Table 5). This may reflect the diverse diets of Timorese farmers, who often eat tubers and purchased rice in addition to maize as a staple food. As there is no safe level of aflatoxin exposure, the widespread detection of aflatoxin-albumin could be a health concern to the population. The study does indicate that when comparing maize contamination and incidence of aflatoxin, there is a significant (P < 0.05) correlation for mothers.

Recalling that the stunting of children in Timor-Leste is among the worst globally^17^, this study also tested the association between the blood levels of children and malnutrition indicators. Based on the relatively low levels of aflatoxin albumin adducts in Timorese children it is not surprising that there is no correlation between aflatoxin albumin levels and stunting. In Tanzania the average levels of aflatoxin albumin adduct (4.7–23.5 pg ml^−1^) were much higher than that reported herein (0.64 pg ml^−1^), and yet the correlation between aflatoxin albumin level and stunting was also non-significant^49^. At much higher levels of aflatoxin albumin concentration (mean 32.8 pg ml^−1^) in Benin and Togo, a correlation was found between aflatoxin albumin concentration and reduced growth in children^37^. Other studies have found a positive correlation between AF in blood and growth^50^ and an inverse relationship between child growth and AF exposure^51^.

The study shows that there is widespread contamination of maize and peanuts by aflatoxins in Timor-Leste. Approximately 36% of maize samples and 26% of peanut samples had detectable levels of aflatoxins. This widespread contamination has led to more than 80% of the adults having detectable levels of aflatoxin-albumin in their blood. Higher levels of aflatoxin contaminated grain and blood levels are found on the south coast where longer wet seasons mean drying grain quickly is very difficult during some harvest times. Although widespread, the absolute levels of aflatoxin in blood are relatively low and are very unlikely to be a major cause of childhood stunting.

## Methods

### Grain samples and analysis

Large samples (8–10 kg) of maize and peanuts were collected from all 13 municipalities of Timor-Leste over the three year period 2013–2015. A total of 296 samples were collected, comprising 190 of maize from the three seasons and 106 of peanuts from the 2013 and 2014 harvest seasons only. Sampling details by crop, season and source are given in Table 1. The maize and peanut samples were randomly acquired from local markets and from seed producers, while maize was also sourced directly from households. Inclusion of seed samples in the study was to compare farmer practice of storing and treating their food grains against seed which is considered to be well treated and hence having reduced levels of fungal contamination. Analyses of variance (ANOVAs) and Pearson correlations throughout the study were computed using GenStat 18^th^ edition (VSN International Ltd., UK).

All samples were cleaned to remove foreign objects, dried to less than 12% moisture and stored in five-litre air-tight containers below 10 °C. Samples collected in 2013 and 2014 were transferred to PT. Angler BioChemLab in Indonesia for analysis of the concentration of the four major aflatoxins (B1, B2, G1 and G2). These analyses were performed by HPLC with Triple Quadrupole Tandem Mass Spectrometry detector (LC-MS/MS) as described in Rosén & Hellenäs^52^ The quantitative analyses were conducted by monitoring Ion Ratio of 2 MRM pairs for each compound and determinations were calculated using matrix-based calibration. Samples collected in 2015 were sent to Australia for the same HPLC analysis at the Peanut Company Australia (PCA) (peanuts) and a laboratory at the University of Sydney (maize). Each sample (250 g) of peanut was extracted by blending with 500 mL of 80% methanol with 4% Na Cl at a high speed for 2 min. The supernatant was collected after filtering through a Whatman No. 1 filter paper. An aliquot of 15 mL was then analysed by HPLC-fluorescence at the PCA Technical Centre using the company’s standard method accredited by National Association of Testing Authorities (NATA, Australia).The detection limit is 2.0 ng mL^−1^.

### Blood collection and analysis

Blood samples were collected by the Ministry of Health across all 13 municipalities of the country within the Timor-Leste Food and Nutrition Survey in 2013^19^. The survey obtained ethics approval from the Ethical Committee of the Faculty of Medicine, University of Indonesia. The Ministry of Health of Timor-Leste also issued an approval letter after a national workshop about the proposed survey was held and the survey protocol had received inputs from relevant stakeholders. Permission was also obtained from local authorities, municipality health offices, and local administrative offices at the respective sub-municipality, villages (*sucos*) and hamlets (*aldeias*). The authors confirm that all research was conducted in accordance with the relevant guidelines and regulations approval. Subjects/respondents and/or their legal guardians were only assessed after they had been informed about the survey procedures and had given their informed consent for study participation. The participation of the subjects in the survey was voluntary. All data was anonymised and treated confidentially and used only for survey purposes^19^. The selection criterion of the population was households with children aged between 6–59 months and their non-pregnant mothers. Approximately 9–10 pairs of children with their non-pregnant mothers were selected per sub-village resulting in 620 blood samples from the 70 selected *aldeias* (village). The number of *aldeias* selected per municipality was according to the size of the population. The blood samples of each *aldeia* were randomly selected through a trained numerator and all blood donators were informed and requested to come to the nearest health center based on an arranged schedule. Three ml of blood sample was taken from each donor.

All blood samples were processed within 6 hours of collection either in the municipality health centre or at the National Laboratory in Dili. Samples were assayed for the presence of aflatoxin - albumin conjugate (aflatoxin attached to human protein via a lysine amino acid covalent bond). The serum was analyzed with a newly developed HPLC-fluorescence method validated by Qian *et al*.^53^. Aflatoxin albumin adducts reflect exposure over a longer period (i.e. 2–3 months)^54^. Aflatoxin B1 (>98% purity), albumin determination reagent bromocreosol purple and normal human serum, were purchased from Sigma Aldrich Chemical Co (St. Louis, MO). Pronase (25kU, Nuclease-free) was purchased from Calbiochem (La Jolla, CA). Protein assay dye reagent concentrate and protein standards were purchased from Bio-Red Laboratories Inc. (Hercules, CA). Authentic ALB-Lys was synthesized as previously described^55,56^. The thawed serum samples were mixed and analyzed for albumin and total protein concentrations using procedures modified as described by Wang *et al*.^57^.

Quality assurance (QA) and quality control (QC) procedures were undertaken during analyses, which included simultaneous analysis of one authentic standard every 10 samples and two QC samples daily. The limit of detection was 0.2 pg mg^−1^ albumin. The serum AFB-Lys level of each sample was adjusted by its albumin content accordingly. The analytical team was blinded as to the study design and the source of sample collections.

### Mother and child anthropometric and haemoglobin measurements

Anthropogenic measurements were taken from the mother and children who gave blood samples also as part of the Timor-Leste Food and Nutrition Survey in 2013^22^. All children aged 0–59 months and their non-pregnant mothers were weighed and measured. The anthropometric assessment was conducted following standardized procedures^58^ by two trained enumerators, either nurses and/or midwives. Standard data were derived for body mass index (BMI) for mother and child, z score for height for age, weight for age and weight for height^58^.

Haemoglobin concentration was measured in children aged 6–59 months and their non-pregnant mothers by trained enumerators (i.e. nurses or midwives). A finger-prick blood sample was obtained using a disposable, sterile lancet and the haemoglobin concentration was measured using HemoCue Hb 201+ System (HemoCue, Ängelholm, Sweden) The HemoCue instruments were calibrated daily using an external standard (i.e. HemoTrol) to check the reliability of the equipment prior to data collection and each team used two machines alternatively. A portable generator was carried to run a chest freezer and centrifuge because many study areas did not have a consistent supply of electricity.

### Spatial analysis

The spatial distribution of aflatoxin contamination in maize and peanut, and levels in mother and child blood was investigated at the level of municipality. There are 13 municipalities in Timor-Leste and the arithmetic average values for aflatoxin contamination for maize and levels on child and mothers blood were calculated for each municipality and compared by one-way ANOVA. Maize and blood samples with undetectable levels were assumed to be zero. There were more than 10 maize samples for all municipalities, except Oe-cusse, which had only 5 samples. The maize contamination data for Oe-cusse were not used in the maps.

## Supplementary information

Supplementary Tables
