# Supplementary material for: Aflatoxin levels in maize and peanut and blood in women and children: The case of Timor-Leste
Source: Sci Rep. 2019 Sep 11;9:13158. doi: 10.1038/s41598-019-49584-1 (PMC6739342; doi:10.1038/s41598-019-49584-1)
Supplement: Supplementary file 1 — Supplementary Tables [file 41598_2019_49584_MOESM1_ESM.pdf]

Supplementary Information

Article Title: **Aflatoxin levels in maize and peanut and blood in women and children: The case of Timor-Leste**

Authors: **Luis de Almeida, Robert Williams, Dirce M. Soares, Harry Nesbitt, Graeme Wright4, William Erskine**

Supplementary Table S1. **The total level of aflatoxin contamination (ng mL^{-1}) and levels of the toxins B1, B2, G1 and G2 in each sample by crop and year.**

Crop	Year	Total Aflatoxin (ng mL^{-1})	B1 Aflatoxin (ng mL^{-1})	B2 Aflatoxin (ng mL^{-1})	G1 Aflatoxin (ng mL^{-1})	G2 Aflatoxin (ng mL^{-1})
Maize	2013	201.1	178.0	23.1	0.0	0.0
Maize	2013	155.6	144.0	11.6	0.0	0.0
Maize	2013	104.2	93.4	10.8	0.0	0.0
Maize	2013	80.3	72.6	7.7	0.0	0.0
Maize	2013	18.1	11.0	0.0	7.1	0.0
Maize	2013	16.6	13.2	3.4	0.0	0.0
Maize	2013	13.0	13.0	0.0	0.0	0.0
Maize	2013	11.9	11.9	0.0	0.0	0.0
Maize	2013	11.0	11.0	0.0	0.0	0.0
Maize	2013	9.5	9.5	0.0	0.0	0.0
Maize	2013	8.6	8.6	0.0	0.0	0.0
Maize	2013	8.1	8.1	0.0	0.0	0.0
Maize	2013	8.0	8.0	0.0	0.0	0.0
Maize	2013	7.2	7.2	0.0	0.0	0.0
Maize	2013	6.7	6.7	0.0	0.0	0.0
Maize	2013	6.5	6.5	0.0	0.0	0.0
Maize	2013	5.8	5.8	0.0	0.0	0.0
Maize	2013	3.4	3.4	0.0	0.0	0.0
Maize	2013	3.2	3.2	0.0	0.0	0.0
Maize	2013	3.0	3.0	0.0	0.0	0.0
Maize	2013	3.0	3.0	0.0	0.0	0.0
Maize	2013	2.9	2.9	0.0	0.0	0.0
Maize	2013	2.6	2.6	0.0	0.0	0.0
Maize	2013	2.5	2.5	0.0	0.0	0.0
Maize	2013	2.4	2.4	0.0	0.0	0.0
Maize	2013	2.1	2.1	0.0	0.0	0.0
Maize	2013	2.1	2.1	0.0	0.0	0.0

Maize	2013	2.1	2.1	0.0	0.0	0.0
Maize	2013	2.0	2.0	0.0	0.0	0.0
Maize	2014	197.9	159.0	9.7	27.7	1.5
Maize	2014	121.9	115.0	6.9	0.0	0.0
Maize	2014	84.8	65.9	18.9	0.0	0.0
Maize	2014	52.3	49.2	3.1	0.0	0.0
Maize	2014	42.6	40.0	2.6	0.0	0.0
Maize	2014	34.2	30.9	3.3	0.0	0.0
Maize	2014	33.2	31.1	2.1	0.0	0.0
Maize	2014	30.6	28.5	2.1	0.0	0.0
Maize	2014	21.8	19.6	2.2	0.0	0.0
Maize	2014	18.4	18.4	0.0	0.0	0.0
Maize	2014	17.4	16.1	1.3	0.0	0.0
Maize	2014	10.1	10.1	0.0	0.0	0.0
Maize	2014	7.9	6.5	1.5	0.0	0.0
Maize	2014	5.5	4.0	0.0	1.6	0.0
Maize	2014	5.0	1.7	0.0	3.3	0.0
Maize	2014	4.3	4.3	0.0	0.0	0.0
Maize	2014	3.8	0.0	0.0	3.8	0.0
Maize	2014	3.8	3.8	0.0	0.0	0.0
Maize	2014	3.4	3.4	0.0	0.0	0.0
Maize	2014	2.4	2.4	0.0	0.0	0.0
Peanut	2014	239.7	210.0	29.7	0.0	0.0
Peanut	2014	161.2	81.8	4.3	71.6	3.5
Peanut	2014	49.9	26.9	2.8	18.2	2.0
Peanut	2014	46.8	23.2	2.5	19.1	2.0
Peanut	2014	14.5	11.9	0.0	2.6	0.0
Peanut	2014	11.2	5.7	0.0	5.5	0.0
Peanut	2014	10.4	10.4	0.0	0.0	0.0
Peanut	2014	8.3	5.6	0.0	2.7	0.0
Peanut	2014	7.7	6.6	1.1	0.0	0.0

Peanut	2014	7.2	7.2	0.0	0.0	0.0
Peanut	2014	6.9	6.9	0.0	0.0	0.0
Peanut	2014	5.4	3.9	0.0	1.5	0.0
Peanut	2014	2.5	2.5	0.0	0.0	0.0
Peanut	2015	365.4	302.2	63.3	0.0	0.0
Peanut	2015	252.6	174.2	20.3	50.7	7.4
Peanut	2015	69.4	46.9	20.7	1.5	0.4
Peanut	2015	46.1	41.2	3.8	0.9	0.2
Peanut	2015	19.3	10.1	0.8	7.9	0.6
Peanut	2015	10.4	6.4	1.5	1.9	0.6
Peanut	2015	4.4	3.1	0.6	0.6	0.0
Peanut	2015	3.2	0.9	0.0	2.3	0.0
Peanut	2015	2.6	2.2	0.1	0.4	0.0
Peanut	2015	2.0	0.6	0.0	1.4	0.0

Supplementary Table S2. Correlation matrix of log_n of child and mother blood aflatoxin albumin levels, mother and child haemoglobin levels and of anthropometric measurements[58] (z score for height for age [haz], weight for age [waz] and weight for height [whz] and body mass index [bmi]) from Ministry of Health survey[22]. 620 data points were available for mothers' data, and 514 for children.

Variate name	Variate #								
Log child aflatoxin 1	1	2	3	4	5	6	7	8	9
Log mother aflatoxin 2	0.42								
Mother bmi 3	0.02	-0.05							
Child bmi 4	-0.02	-0.01	0.06						
Mother haemoglobin 5	-0.06	-0.01	0.08	0.04					
Child haemoglobin 6	0.06	-0.02	0.07	-0.15	0.19				
Child Z bmi 7	0.02	-0.02	0.10	0.88	0.05	0.04			
Child Z haz 8	-0.08	-0.05	0.12	-0.03	-0.01	-0.06	-0.16		
Child Z waz 9	-0.06	-0.06	0.18	0.55	0.02	-0.03	0.49	0.77	
Child Z whz 10	0.01	-0.04	0.14	0.89	0.04	0.04	0.96	0.03	0.64
